# 780 Injury to Intervention: Is There Another Opportunity to Improve Burn Care?

**DOI:** 10.1093/jbcr/irad045.255

**Published:** 2023-08-29

**Authors:** William L Hickerson, Jeffrey E Carter, John S Friedstat, James H Holmes, James Hwang, Steven A Kahn, Herbert A Phelan, Alisa Savetamal

**Affiliations:** LSUHSC, Memphis, Tennessee; University Medical Center New Orleans & LSUHSC, River Ridge, Louisiana; MGH, Boston, Massachusetts; WFSOM, Winston-Salem, North Carolina; UAB, Birmingham, Alabama; The South Carolina Burn Center/Medical University of South Carolina, Charleston, South Carolina; LSUHSC New Orleans Department of General Surgery, New Orleans, Louisiana; Yale New Haven Health, Bridgeport Hospital, Bridgeport, Connecticut

## Abstract

**Introduction:**

The treatment of burn-injured patients requires early intervention to prevent adverse events such as local or systemic infection, increased mortality, or exacerbation of other homeostatic processes. Early intervention also reduces delays in treatment, return to work, and the related costs of hospitalization, wound care, and pain control. The purpose of this study was to evaluate potential delays in treatment and the associated costs compared to those seen with more accurate diagnosis and treatment on admission.

**Methods:**

A prospective, IRB-approved study was undertaken with nine burn centers in nine states and three ABA burn regions. Data points included times of injury, admission, and surgery. Inclusion criteria included: life expectancy > 6 months, thermal burn mechanism (flame, scald, or contact), and minimum burn wound size for adult or pediatric patients of 0.5% TBSA. Data was collected by onsite research staff and confirmed during periodic monitoring visits. Avoidable inpatient days (AID) was defined as the time of admission to the time of surgery, and avoidable treatment delays (ATD) as the time of injury to the time of surgery. The average daily bed costs were defined as $7,554 from recent real-world published data.

**Results:**

One hundred thirty-eight (138) patients in this study were admitted and treated with surgical intervention for full thickness burn injuries from 2017 to 2022. The total AID was 506 (median 3 days, range 0-9, mean 4, 25%tile 3, 75% 4). The total ATD was 733 days for 138 patients (median 5 days, range 0-9, mean 5, 25%tile 4, 75%tile 6). The median AID cost savings were calculated at $22,662 per patient had surgery been indicated and performed within 24 hours of admission. The median ATD cost savings were $30,216 per patient had surgery been indicated and conducted within 24 hours of injury or evaluation at an emergency room or burn center. The total savings for 138 patients whose surgery had been determined necessary and performed within 24 hours of the burn would have been $4,169,808 as opposed to having surgery indicated and completed within 24 hours of admission, $2,084,904.

**Conclusions:**

If early diagnosis of burn injuries requiring surgery could be obtained within 24 hours of injury or admission, facilities and patients would benefit from reduced treatment delays and inpatient care costs. Our study was limited to patients undergoing surgery and those with non-healing wounds. An additional benefit could also be found in patients with healing wounds; if a confident diagnosis could be made earlier, such patients could be discharged rather than spending excess time as inpatients. Further studies are in progress with larger populations and more burn centers to better determine benefits for these patients.

**Applicability of Research to Practice:**

We must understand the economic impact of our clinical decisions to pursue research initiatives that are clinically significant to our patients and financially viable to the institutions that support burn care.

